# A Qualitative Exploration of the Support Needs of Individuals With Type 2 Diabetes and Disordered Eating Behaviours

**DOI:** 10.1155/jdr/7718760

**Published:** 2026-07-29

**Authors:** Emma Reid, Talitha Best, Alicia Carter, Melissa Oxlad

**Affiliations:** ^1^ NeuroHealth Lab, School of Health, Medical, and Applied Sciences, Central Queensland University, Brisbane, Australia, cqu.edu.au; ^2^ The Centre for Health Equity in Regional and Remote Communities, School of Health, Medical, and Applied Sciences, Central Queensland University, Cairns, Australia, cqu.edu.au; ^3^ School of Psychology, Adelaide University, Adelaide, Australia, adelaide.edu.au

## Abstract

**Aims:**

We aim to explore the support needs of individuals living with type 2 diabetes and disordered eating behaviours.

**Methods:**

We conducted semistructured interviews with adult participants living with type 2 diabetes and self‐identified disordered eating behaviours recruited from an Australian allied health service. Data were analysed through reflective thematic analysis.

**Results:**

Ten adults with type 2 diabetes (seven female, three male; mean age 66.3 ± 8.97, range: 52–80) were interviewed. Four themes are presented related to participants′ support needs: (1) the need for increased information about the interactions between food, eating behaviours and diabetes and self‐management; (2) a desire for increased time with knowledgeable health professionals who provide tailored person‐centred diabetes care and support; (3) mental health services integrated into diabetes care are desired due to relationships between mental health, disordered eating behaviours and diabetes management; and (4) family, peers and community can offer invaluable support, but stigma can be a barrier to support seeking.

**Conclusions:**

Individuals living with T2DM and disordered eating behaviours report diverse support needs including tailored informational support and emotional support from health professionals, family and peers. Although further research is required to develop and evaluate the implementation of specific interventions and care pathways, health professionals should endeavour to provide adequate time and resources for personalised education, and to promote and facilitate appropriate mental health and peer supports.

## 1. Introduction

Type 2 diabetes mellitus (T2DM) affects approximately 508 million people worldwide and is characterised by impaired insulin secretion and/or insulin resistance, resulting in high blood glucose levels [1, 2]. With global prevalence of T2DM predicted to more than double by 2050 [1], there is an increasing need to address the complex factors contributing to its development and progression. Disordered eating behaviours may be one such factor.

Disordered eating and eating disorders both appear common among individuals with T2DM, with a systematic review involving 6527 participants reporting a binge eating disorder (BED) prevalence of 1.2%–25.6% [3], with rates of disordered eating attitudes and behaviours appearing even higher at 29.6% [4]. Both diagnosed eating disorders (i.e., BED and bulimia nervosa [BN]) and nonclinical measures of disordered eating behaviours (i.e., binge eating, restricting food and purging) have been independently associated with greater insulin resistance and increased risk of T2DM [5–8]. Disordered eating consequently appears to be an important target for intervention. However, the support needs of individuals experiencing disordered eating alongside T2DM requires further investigation.

Engaging individuals who are to be targeted by any future intervention is a core step in complex intervention development [9], and qualitative research is well suited to this task, allowing us to more richly understand the experiences that any future intervention aims to influence [10]. However, there has been minimal qualitative research conducted exploring the support needs of those with T2DM and disordered eating. One previous study involved interviews with 21 women with T2DM or prediabetes recruited from a BED treatment program [11, 12]. These women identified the need for better synergy between diabetes and BED treatment, more individualised treatment plans and for health practitioners to be nonjudgmental and better understand the difficulties of managing both conditions [11, 12]. Another study included 20 adults (13 women and 7 men) with T2DM and BED or clinically significant binge eating [13]. Participants wanted to better understand the biomedical mechanisms of binge eating and for healthcare providers to directly ask about binge eating behaviours [13].

Although these studies provide insight into the support needs of those with binge eating and T2DM, their focus on binge eating means the support needs of individuals with other disordered eating behaviours, or those at subclinical levels, are not yet known. We aim to explore the support needs of individuals with T2DM who engage in any form of disordered eating.

## 2. Methods

### 2.1. Design

We utilised a qualitative research design involving semistructured interviews. A semistructured interview guide was developed based on the existing T2DM and disordered eating literature as well as the authors′ previous clinical experience as psychologists providing psychological support to individuals with T2DM and/or disordered eating. In line with recommendations [14], an initial interview guide was developed and field‐tested, with refinements made based on early interviews. Example interview questions included ‘Within the Australian healthcare system as a whole, how well do you think people with T2DM and disordered eating are supported?’ and ‘What would you like health professionals to know about disordered eating and T2DM?’ The full semistructured interview guide is presented in Table S1. The Central Queensland University Human Research Ethics Committee approved this research (Approval Number: 24860), and we report the study according to Consolidated Criteria for Reporting Qualitative Research guidelines (COREQ see Table S2) [15].

### 2.2. Recruitment and Sample

We recruited participants from an interprofessional allied health service that supports individuals with or at risk of T2DM located in a region of South East Queensland, Australia associated with high levels of health inequity [16]. Eligibility criteria for the current study included having a diagnosis of T2DM or prediabetes, being over the age of 18 and self‐identifying to struggle with one or more of the following plain‐English descriptions of common disordered eating behaviours: eating due to stress or other emotions, feeling out of control with eating, avoiding certain types of foods or feeling guilty for eating them, skipping meals or not eating enough or engaging in unhealthy weight loss tactics. As many potential participants may not be familiar with the clinical terms ‘disordered eating’ or ‘eating disorder,’ we did not include these terms in recruitment material. Additionally, although initial eligibility criteria included individuals with prediabetes, only one such individual was recruited, likely due to the characteristics and focus of the recruitment site [16]. In order to avoid misrepresenting the support needs of individuals with T2DM, the decision was made to exclude this participant from the current study.

Participants were recruited using a clinic‐wide email newsletter and in‐clinic posters.

Individuals who completed an expression of interest were contacted by telephone or by email by the first author. Eligible participants were provided with an information sheet, and interview times were scheduled via telephone or email. Verbal consent was formally obtained prior to interview commencement, with consent recorded and documented.

### 2.3. Data Collection

Interviews were conducted between December 2024 and April 2025 via telephone or Zoom teleconferencing. A researcher not otherwise affiliated with the study or recruitment site conducted the interviews to reduce bias and any risk of coercion, given that the first author is a health psychologist working at the recruitment site. The interviewer was a woman with a background in psychology and experience in qualitative research. All interviews were audio‐recorded and transcribed verbatim.

In line with qualitative research recommendations to increase collaboration and credibility [17], participants were provided with a copy of their transcripts via email and given an opportunity to provide member reflections through reply email or telephone call. One participant requested that additional information regarding their difficulties accessing mental health care be added to their transcript. No other participants requested any additions or changes to their transcript. Participants received a 20 AUD gift card for their time.

Ten interviews were included in the current study. This was an exploratory study with a narrow aim, specific sample and high quality of dialogue which used a cross‐case analysis strategy (coding and analysing each interview separately before then comparing them) to develop shared themes [18]. As such, sample size was considered adequate based on the information power model [18]. Interviews ranged from 25 to 50 min in duration (M = 36.6 min).

### 2.4. Data Analysis

We chose a reflexive thematic analysis methodology due to its theoretical flexibility, focus of meaning‐based themes and central acknowledgment of researcher positionality and reflexivity [19]. We analysed the data following guidelines by Braun and Clarke [20] using a largely inductive approach with an essentialist theoretical perspective (where participants′ language is taken to directly reflect their perceptions, experiences and realities) and experiential orientation (which seeks to understand participants′ perceptions and experiences). Initial familiarisation with the data occurred through review of the transcripts and discussions with the interviewer. All transcripts were reviewed by the first author and a subset by all other members of the research team. The first author then completed initial coding using NVivo 14 software and generated preliminary codes. Codes were reviewed and refined by all members of the research team. Preliminary themes were then developed by the first and last authors, which were subsequently cross‐checked and reconciled by the full research team until consensus regarding final themes was agreed upon.

We acknowledge how our positionality may influence our research. Regular peer‐debriefing and self‐reflexivity practices occurred through the data collection and analysis processes. We are all women with a background in health psychology and living without T2DM. The first author is a health psychologist employed at the recruitment site to provide psychological services to individuals with disordered eating and T2DM, and may thus be considered an insider with some pre‐existing knowledge of the experiences explored. Therefore, we enlisted an independent researcher to facilitate interviews to reduce the influence of researcher positionality on data collection. Additionally, codes and themes were reviewed and refined by all researchers, with all other members of the research team considered outsiders to the research.

## 3. Results

### 3.1. Participants

Ten individuals with T2DM were interviewed (seven female, three male; mean age 66.3 ± 8.97, range: 52–80). Nine participants described their cultural background as Australian and one as Aboriginal Australian. Three participants were married, three were widowed and four divorced or single. The majority (70%) of participants had been living with diabetes for more than 6 years. Self‐described disordered eating behaviours included binge eating, emotional eating and restrictive eating behaviours. Full participants′ demographics are presented in Table 1.

**Table 1:** Demographic characteristics of participants (*n* = 10).

Demographics	*n* (%)
Gender	
Female	7 (70%)
Male	3 (30%)
Cultural background (self‐described)	
Australian	9 (90%)
Aboriginal and/or Torres Strait Islander	1 (10%)
Marital status	
Married or de facto	3 (30%)
Widowed	3 (30%)
Single	3 (30%)
Divorced	1 (10%)
Highest level of education	
Year 9	2 (20%)
High school	1 (10%)
Certificate	3 (30%)
Diploma	1 (10%)
Associate diploma	1 (10%)
Bachelor′s degree	1 (10%)
Not reported	1 (10%)
Family history of diabetes	
Yes	6 (60%)
No	4 (40%)
Time since diagnosis of type 2 diabetes	
< 1	1 (10%)
1–6 years	1 (10%)
6+ years	7 (70%)
Unknown	1 (10%)

### 3.2. Themes

**Figure 1:**
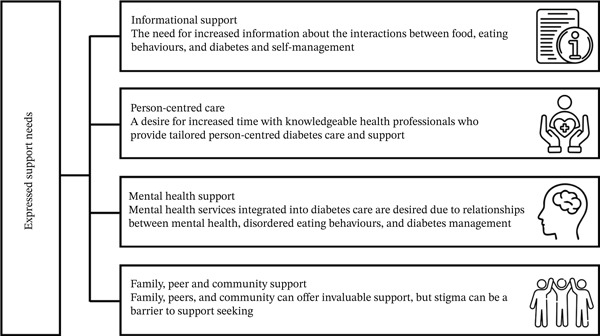
Thematic map.

There were four themes generated in relation to participants support needs (Figure 1).

#### 3.2.1. Theme 1: The Need for Increased Information About the Interactions Between Food, Eating Behaviours and Diabetes and Self‐Management

Participants reported not being provided with adequate information about diabetes broadly, nor about interactions between eating behaviours and diabetes. They explained that although they had received broad behavioural instruction on what they should and should not do as part of diabetes self‐management, there had been insufficient attention to the causes of high blood glucose levels or the ‘why’ behind behavioural advice.

If I go in and my blood sugar′s 20 I get told, ‘oh you know, you′ve got to bring it down, it′s really high.’ But not why? How did you get that high and the dangers that having it that high. That′s never been addressed. (Participant 7, female).

Insufficient education regarding the interaction between eating behaviours and diabetes self‐management was noted to be widespread. One participant, when speaking about her experience as part of a group diabetes lifestyle management program, described how health professionals had not previously provided this information to most participants in her group.

There was about eight of us in the group, and I was absolutely mortified that the others who were there had no idea how food affected them… I just sat there and I′m thinking, why has not your doctor told you this? Why have not you been to see people, you know? They had no idea whatsoever… And I′m thinking well that′s the doctor′s fall down there. (Participant 10, female).

Participants also expressed wanting more specific information regarding eating behaviours and diabetes self‐management, including meal regularity and timing, portion sizes and how specific foods impacted hunger/fullness and blood sugar levels. When health professionals did not provide this information, participants sought it through books, magazines or online searches: ‘About food, food related and what could I possibly eat throughout the day to keep my levels low…Yeah, that′s where I was like what can I do? So I′ve gone out and sourced things myself’ (Participant 2, female).

Some participants reported greater trust in information provided by health professionals than from online sources; ‘I kind of trust the information I′m given, which is very good. Other than looking it up on the Internet and getting all sorts of results’ (Participant 11, male). Others described thinking that certain health professionals did not fully understand diabetes, or that advice was too generalised and not tailored to their specific needs and circumstances. Participants expressed a need for person‐centred diabetes care.

#### 3.2.2. Theme 2: A Desire for Increased Time With Knowledgeable Health Professionals Who Provide Tailored Person‐Centred Diabetes Care and Support

Participants described health professionals′ advice around diet and eating behaviours as overly generic, not personalised to their needs or tastes or not applicable to ‘real life’: ‘I′ve been to dietitians before and they all paint with the same brush’ (Participant 3, female).

At times, participants also described feeling as though they were being bounced from service to service, never having the opportunity to ask the questions or receive the information or support they desired.

The one at the hospital, very limited [information about eating behaviours]. They sort of just gave you your medication. Tested your levels and gave you medication and sort of sent you on your way. I got brought back for a checkup 6 months later or whatever it was. They adjusted me medication and then, catch you later… I was sort of thrown straight back to me GP and said let them deal with it. (Participant 9, male).

Participants noted having insufficient access to, or time with, health professionals. Limited time with health professionals, perceived to be a systemic issue, was a barrier to discussing issues at participants′ own pace.

They′ve only got a matter of time to see you too. So the dietician would be going ‘blah blah blah.’ And you go ‘oh hang on wait on. I′m only still back at the first part.’ And it wasn′t their fault. It was the time they were allotted. (Participant 8, female).

This participant went on to describe the positive impact of having more time with health professionals and the opportunity to build relationships, which made them feel ‘just as important as the next person’ (Participant 8, female).

I got more education here than I ever have through doctors and dietitians outside because they do not have time to spend with you. I think it′s a lot to do with the crew here… They all, when I come in, everybody′s got a smile. Everybody′s ‘hey [participant′s name], how you going?’ and it′s just like coming home. (Participant 8, female).

Another participant also expressed how person‐centred care broke down barriers to asking questions or for more support with their diabetes and disordered eating behaviours: ‘Yeah, and it′s, I′m not afraid to walk in there and you know, ‘hey, I need some help with this.’’ (Participant 11, male).

I got more education here than I ever have through doctors and dietitians outside because they do not have time to spend with you. I think it′s a lot to do with the crew here… They all, when I come in, everybody′s got a smile. Everybody′s ‘hey [participant′s name], how you going?’ and it′s just like coming home. (Participant 8, female).

Another participant also expressed how person‐centred care broke down barriers to asking questions or for more support with their diabetes and disordered eating behaviours: ′Yeah, and it′s, I′m not afraid to walk in there and you know, ‘hey, I need some help with this.’′ (Participant 11, male).

Having an open dialogue with health professionals was important in enabling conversations about disordered eating in diabetes care. When asked if they had ever discussed their emotional eating with a health professional, one participant explained that they had not due to feelings of shame. They later went on to express that they wished health professionals would initiate conversations and explicitly ask about disordered eating.

Oh, with the overeating I think it should have been a question at some stage through seeing a diabetic nurse. And it′s going you know like ‘do you under eat?,’ ‘do you over eat?,’ that kind of thing. Just, or even referred to a dietician so that they can. Somebody needs to ask that question because that has never been asked. (Participant 7, female).

Acknowledging the emotional components of eating behaviours in diabetes whether that be diabetes‐related shame or the impacts of other mental health concerns was important to participants.

#### 3.2.3. Theme 3: Mental Health Services Integrated Into Diabetes Care Are Desired due to Relationships Between Mental Health, Disordered Eating Behaviours and Diabetes Management

Many participants described mental health as considerably influencing their diabetes management, and emotional distress as triggering certain disordered eating behaviours. They expressed a desire for mental health support to be embedded in diabetes care.

Because very much my mental health is a big one in actually keeping my type 2 diabetes in control. So, even when I know exactly what I′ve got to do, if mental health is out the window for a month or two months, it′s yeah, it′s just, I do not know, depressing, I suppose. That, yeah, that I cannot do both. (Participant 11, male).

Participants noted that although health professionals such as doctors or dietitians may identify the impact of mental health on diabetes management, they may not be best placed to address this. One participant described their experience of being referred to a dietitian for support around emotional eating but thinking this was not what was needed to address the psychological components of their behaviour.

But then my doctor said, well, she [dietitian] should know about emotional eating… Hmm, I think my dietitian, I think [she] was a bit perplexed by this. I do not think she thought she was the person to sort that out. Because it′s, and I did not really think so either… It′s a mental thing. I know it′s a mental thing, and she′s not going to get into my head. (Participant 5, female).

While some participants noted an increase in awareness and promotion of mental health support, others emphasised that awareness still needed to be improved, particularly in diabetes contexts. Participants also described the need to have mental health support integrated as part of diabetes care, as mental health professionals in general services may not have sufficient understanding of diabetes to provide the desired support.

Probably a little bit more publicity would be cool. I did not know that they had a psychologist on staff who dealt with psychology with reference to this; this is all within the diabetic′s section. Um, so that I could have been talking to her psychologically and she would understand diabetes because I was speaking to a psychologist outside the diabetic system and that they did not understand it as much. (Participant 4, female).

However, participants also highlighted the importance of being able to discuss mental health holistically, not just in relation to diabetes. One participant expressed how they needed to address their eating disorder and other mental health concerns before being able to attend to diabetes directly. They noted that ‘until I got my head straight, my diabetes wasn′t either’ (Participant 8, female) and how improvements in their mental health led to improvements in their diabetes management.

With the eating disorder, mine was totally out of control as I′ve told you. But actually talking to somebody about it. So my psychologist outside. Talking to her about it, actually brought things back into perspective, and helped me start sorting everything out… So, I think that′s when everything started to click was when my head became more stable and I could actually take in what people were saying to me. (Participant 8, female).

In addition to the benefits of formal psychological support, participants also explained the benefits of support from family or peers in managing mental health and eating behaviours in diabetes.

#### 3.2.4. Theme 4: Family, Peers and Community Can Offer Invaluable Support, but Stigma Can Be a Barrier to Support Seeking

Participants described the role family members played in supporting them with mental health, eating behaviours and diabetes and related complications. Support from loved ones, such as partners, was appreciated and valuable: ‘I′m very grateful for it. He′s [husband] not perfect. He annoys the hell out of me sometimes. But he is there when I need it’ (Participant 4, female). Participants also expressed that their families, in particular immediate family members who may witness the day‐to‐day struggle with diabetes, eating and blood sugar levels, needed more support; ‘I think there needs to be a greater emphasis of how the other person can cope’ (Participant 1, male).

Additionally, participants stated that, at times, stigma was a barrier to reaching out for family support. One participant explained how sharing difficulties was not the norm in their family, so they thought they needed to address mental health and emotional eating independently.

It′s like I still cannot talk to a lot of people about what′s happening because, you know, it′s my business and I have to sort it out. Even my own family, I will not discuss it with them because it′s not their problem. It′s my problem. (Participant 7, female).

This participant, who was an Aboriginal Australian, also discussed the important role community, particularly elders, could play in supporting people with managing their diabetes and eating behaviours.

And whether you start with kids or whether you start with the elders. I do not know but. You know. You gotta start somewhere and then follow it down. It′s like I grew up learning things from my grandmother and I still do today because Granny said you know you do XYZ. And I think that′s what′s missing in today′s society, that there′s no pass it down the family, that sort of training has gone. (Participant 7, female).

In addition to family support, participants discussed the benefits of peer support. They expressed that interacting with others experiencing similar concerns as part of a group diabetes lifestyle management program was beneficial in normalising difficulties and providing practical tips and guidance.

They have an 8‐week course and that gets you in a group of 10 or 12 people who are either asking all the same questions or in the same boat as yourself. And it does not then become ‘it′s just me fighting.’ It′s like you end up with a friendship group even though you do not know them outside of there. But, yeah, it kind of normalised it a bit for me and that was, yeah, kind of a friendly place, I suppose, where we have learned a lot of information and even things like checking yourselves, you know, some people are doing it a different way and you′ll go ‘oh, that′s a good idea.’ So, I′ve found a lot of really good in that rather than reading documentation given to me by a doctor. (Participant 11, male).

Participants benefited from support from family and peers, alongside the support from a variety of health professionals.

## 4. Discussion

We aimed to explore the support needs of individuals with T2DM who engage in disordered eating behaviours. Our participants identified the need for greater informational support, for person‐centred diabetes care, for mental health integrated into diabetes care and family, peer and community support.

Receiving insufficient information about diabetes and eating behaviours was a common issue among our participants. When received, participants often described information to be overly generic and not tailored to individual circumstances. The desire for greater information is consistent with previous qualitative research on binge eating and T2DM, where participants wanted to know more about the biomedical underpinnings of binge eating [13], and described diabetes education that incorporated binge eating as highly beneficial [11]. Informational support, including nutrition‐specific information, is also a well‐known need within the broader diabetes literature across both type 1 diabetes (T1DM) and T2DM [21], as well as in other chronic disease populations where self‐management may involve a significant dietary component, such as inflammatory bowel disease [22]. However, when health professionals do not meet individuals′ informational support needs, they may naturally seek information from other sources such as online, as described by our participants. This comes with potential risks as online information may be incomplete [23] or unreliable [24]. There is thus a need for high‐quality, personalised education regarding the interaction between diabetes and disordered eating, including codesign and evaluation of such resources. Educational material can also be used to promote more positive relationships with food and body image, and information delivered by health professionals can be tailored to increase strength‐based resources. However, health professionals cannot deliver such tailored education surrounding disordered eating if they are not asking about these behaviours.

In both the current study and previous research [13] participants were rarely asked about disordered eating during diabetes care despite indicating that direct screening would have been welcomed and valued. Although little is known about health professionals′ views towards screening for disordered eating in T2DM, in the T1DM literature, health professionals have expressed a lack of confidence to directly ask about disordered eating behaviours [25]. A perceived lack of knowledge about disordered eating influenced this low confidence, with health professionals fearing they would be overstepping, would receive negative reactions, and lack referral pathways should disordered eating be identified [25]. While dedicated research is required, similar barriers may plausibly exist for health professionals supporting those with T2DM. Additional professional development for health professionals working in this space, including training on validated screening tools such as the Diabetes Eating Problem Survey‐10 [26], may help to address barriers regarding knowledge and confidence. Dialogue tools, such as those cocreated for discussions regarding binge eating in individuals with T2DM [27], may also support health professionals′ confidence and the provision of such conversations in a supportive and nonstigmatising manner, with further research needed to evaluate the implementation of such tools. However, the noted lack of referral pathways to appropriate mental health support [25] indicates a systemic barrier, as also highlighted by participants in the current study.

Our participants noted the direct link between disordered eating and mental health concerns, and the need for greater mental health support. Within T2DM qualitative literature, participants have similarly noted an absence of emotional or mental health support, both in relation to emotional eating and to overall mental well‐being [28, 29]. Both national [30] and international [31] T2DM management guidelines recommend that mental health screening and support be integrated into diabetes care. However, this is not always the case in practice, as noted in our study. Inadequate access to mental health support and treatment coverage is a well‐known systemic issue globally [32] and a national issue in Australia where this research was conducted [33]. Identified barriers to mental health care include navigation of system complexity and lack of available and affordable services, including specialist services as would be required for this population [34]. Greater emphasis must be placed on incorporating mental health professionals into diabetes teams or ensuring adequate and navigable referral pathways exist to diabetes‐knowledgeable professionals.

Outside of the healthcare system, our participants also noted the role of support from family and peers. Family support is an important element in facilitating diabetes self‐management, with self‐management interventions incorporating a family support component shown to improve health behaviours, self‐efficacy, psychological well‐being and glycaemic control [35]. However, as noted in our own and previous research [13], individuals are sometimes reluctant to disclose disordered eating to family due to fear of judgement and shame. Unfortunately, eating disorder symptoms are associated with high levels of shame [36], which may be further exacerbated for those also living with T2DM given the stigma surrounding the condition. Indeed, diabetes stigma (self‐stigma and experiences of being treated differently) has been found to be associated with more disordered eating behaviours (binge eating and eating as a coping strategy) among those with T2DM [37]. Peer support groups may play a role in addressing stigma and shame regarding disordered eating [38]. In our study, a participant described how group peer support helped to normalise struggles with eating and diabetes. As peer support is also known to improve self‐management and self‐efficacy for individuals with T2DM [39], its integration into diabetes care may have broad benefits. Future research is required to specifically evaluate the impact of peer support groups on disordered eating outcomes within this population.

### 4.1. Strengths and Limitations

Given that a predominance of broader disordered eating research has been conducted with younger adults or adolescents, our findings from middle‐aged and older adults provide important insights into the support needs of this underrepresented group. Additionally, all participants were recruited from a diabetes clinic in a region associated with high levels of health inequity [16]. There are known associations between socioeconomic status and T2DM outcomes [40], and the insights from our participants further enhance awareness about the perceptions and experiences of people living in areas with socioeconomic disadvantage.

However, our findings must also be interpreted within the context of study limitations. Given the demographics of the study population, our findings may not be representative of younger or newly diagnosed individuals, or of individuals from other socioeconomic backgrounds. Our sample was also of limited cultural diversity, and may not represent the experiences of individuals from diverse cultural backgrounds. Although one participant provided valuable insight into the unique support needs of Aboriginal Australians, including the importance of involving elders and communities, further research is needed to fully understand the unique needs of Aboriginal Australians with T2DM and disordered eating, as well as those from other cultural backgrounds.

## 5. Conclusion

Individuals living with T2DM and disordered eating behaviours report diverse support needs, including informational support tailored to their individual needs and circumstances and emotional support from health professionals, family and peers. Future research is required to develop and evaluate specific interventions to address these support needs. Such interventions may include educational resources, implementation research for integrating disordered eating screening into routine diabetes care, evaluation of peer support programs on disordered eating and diabetes outcomes, and the development and implementation of integrated care pathways for diabetes and mental health support. Although systemic barriers present challenges, health professionals and services should endeavour to provide adequate time and resources for personalised education, and to promote mental health and peer support. Greater support for individuals with T2DM and disordered eating may help to improve both physical and mental health outcomes, ultimately improving quality of life and reducing disease burden.

## Author Contributions

**Emma Reid:** conceptualization, methodology, data curation, formal analysis, visualization, project administration, writing – original draft. **Talitha Best:** conceptualization, methodology, formal analysis, validation, supervision, writing – review and editing. **Alicia Carter:** conceptualization, methodology, formal analysis, validation, supervision, writing – review and editing. **Melissa Oxlad:** conceptualization, methodology, formal analysis, validation, supervision, writing – review and editing.

## Funding

This study was supported by the Australian Government Research Training Program (RTP) Scholarship (10.82133/C42F-K220). Open access publishing facilitated by Central Queensland University, as part of the Wiley—Central Queensland University agreement via the Council of Australasian University Librarians.

## Disclosure

All authors have read and approved the final version of the manuscript. Emma Reid had full access to all of the data in this study and takes complete responsibility for the integrity of the data and the accuracy of the data analysis.

## Conflicts of Interest

The first author is an employee at the primary recruitment site. The other authors declare no conflicts of interest.

## Supporting Information

Additional supporting information can be found online in the Supporting Information section.

## Supporting information


**Supporting Information 1** Table S1 presents the full semistructured interview guide utilised for this study.

**Supporting Information 2** Table S2 presents the Consolidated Criteria for Reporting Qualitative Research checklist.

## Data Availability

The data that support the findings of this study are available from the corresponding author upon reasonable request.
