# Supplementary material for: A Qualitative Exploration of the Support Needs of Individuals With Type 2 Diabetes and Disordered Eating Behaviours
Source: J Diabetes Res. 2026 Jul 29;2026:7718760. doi: 10.1155/jdr/7718760 (PMC13420349; doi:10.1155/jdr/7718760)
Supplement: Supplementary file 1 — Supporting Information 1 Table S1 presents the full semistructured interview guide utilised for this study. [file JDR-2026-7718760-s001.docx]

**Supplementary Material**

**Table S1.**

*Semi-structured Interview Guide*

| Primary questions | Optional Prompts |
| --- | --- |
| Demographics *(asked in conversational style)*   - Age - Gender - Cultural background - Relationship status - Highest level of education - Family history of diabetes - type 2 diabetes diagnosis |  |
| Can you please tell me a little about your experience of living with type 2 diabetes? | - How long have you been living with type 2 diabetes? - What did you think when you were diagnosed with type 2 diabetes? - How did you feel when you received the diagnosis? - What have been your experiences with food after being diagnosed with type 2 diabetes? |
| Please tell me a bit about your overall relationship with food? | - Are you satisfied with your eating patterns? - Do you have any rules around food? - Do you have any food you don’t allow yourself to eat? - Do you have any food you feel out of control with? |
| What concerns or challenges, if any, do you have around food and eating? |  |
| What does the term disordered eating mean to you? | - Disordered eating behaviours are very common, and many people may have experienced them at some point in their life. They might include things such as: emotional eating or binge eating; extreme dieting, fasting or skipping meals; avoiding types of foods or food groups for non-medical reasons; or using unhealthy weight loss tactics. |
| Have there been times when you’ve engaged in any of these disordered eating behaviours? |  |
| How have these disordered eating behaviours impacted you? | - When did these disordered eating behaviours first begin for you? - What do you think may have contributed to the development of these disordered eating behaviours? |
| Which came first for you, the disordered eating behaviours or the type 2 diabetes diagnosis? |  |
| How, if at all, do you think these disordered eating behaviours impact your type 2 diabetes management? | - How, if at all, do you think these eating behaviours impact your physical health? - How, if at all, do you think these eating behaviours impact your mental health? |
| How, if at all, do you think living with type 2 diabetes impacts your eating behaviours and relationship with food? |  |
| Have you ever discussed these eating behaviours with a health professional? | - If yes: What encouraged you to do this? What was the experience like for you? - If no: What has stopped you from doing this? What would have helped you feel comfortable to raise this with a health professional? |
| What support, if any, have you received for these eating behaviours? | - What has been most helpful? - What has been the most challenging |
| Within the Australian healthcare system as a whole, how well do you think people with type 2 diabetes and disordered eating are supported? |  |
| What would you like health professionals to know about disordered eating and type 2 diabetes? |  |
| We plan to design an intervention to better support people living with type 2 diabetes and disordered eating behaviours. What supports, services, or programs would have been helpful for you. What supports do you wish existed? |  |
